# A Molecular Landscape of Mouse Hippocampal Neuromodulation

**DOI:** 10.3389/fncir.2022.836930

**Published:** 2022-05-06

**Authors:** Stephen J Smith, Mark von Zastrow

**Affiliations:** ^1^Allen Institute for Brain Science, Seattle, WA, United States; ^2^Departments of Psychiatry and Pharmacology, University of California, San Francisco, San Francisco, CA, United States

**Keywords:** hippocampus, mouse, neuromodulation, GPCR (G protein-coupled receptor), ion channel, transcriptome, single-cell RNA-Seq

## Abstract

Adaptive neuronal circuit function requires a continual adjustment of synaptic network parameters known as “neuromodulation.” This process is now understood to be based primarily on the binding of myriad secreted “modulatory” ligands such as dopamine, serotonin and the neuropeptides to G protein-coupled receptors (GPCRs) that, in turn, regulate the function of the ion channels that establish synaptic weights and membrane excitability. Many of the basic molecular mechanisms of neuromodulation are now known, but the organization of neuromodulation at a network level is still an enigma. New single-cell RNA sequencing data and transcriptomic neurotaxonomies now offer bright new lights to shine on this critical “dark matter” of neuroscience. Here we leverage these advances to explore the cell-type-specific expression of genes encoding GPCRs, modulatory ligands, ion channels and intervening signal transduction molecules in mouse hippocampus area CA1, with the goal of revealing broad outlines of this well-studied brain structure’s neuromodulatory network architecture.

## Introduction

The primary function of every neuron is communication with other neurons. Though many (or all) neurons also communicate with glial cells, and some also serve as sensory transducers or signal directly to muscles or glands, most present thinking about the neuronal mechanisms of animal perception, memory, cognition, and behavior revolves around neuron-to-neuron communication. All neurons share one basic communication mechanism: they send messages by secreting diffusible ligands from one neuron to activate receptors displayed on the surface membrane of a second, target neuron. These receptors typically act to govern the ion channels that establish the target cell’s electrical excitability, activity and synaptic strength. Most individual neurons communicate in this fashion with a relatively small number (several to several hundred) of other individual neurons. This simple notion neatly encapsulates a way to grapple the vast complexities that arise as individual neuron-to-neuron connections iterate through extended neuronal networks that may comprise billions. Neurons are, however, extremely diverse. They are dizzyingly diverse in their morphologies, the messenger ligands they secrete, the receptors they display, and their ion channel complements. It now appears, however, that it may be possible to corral all these varied dimensions of neuronal diversity into a unified “neurotaxonomy,” where knowing a given neuron’s “type” allows strong prediction of that neuron’s morphology, and thereby it’s opportunities to connect to particular other neurons, as well as the particulars of that neuron’s molecular signaling and electrogenic machinery (Petilla Interneuron Nomenclature Group et al., 2008; Fishell and Tamas, 2014; Kepecs and Fishell, 2014; Oh et al., 2014; Cadwell et al., 2017; Zeng and Sanes, 2017; Tasic et al., 2018; Zeisel et al., 2018; Fishell and Kepecs, 2019; Gouwens et al., 2019; Huang and Paul, 2019; Ren et al., 2019; Miller et al., 2020; Gala et al., 2021; Yao et al., 2021; Campagnola et al., 2022). Here we explore this premise as it pertains to neuromodulatory signaling in region CA1 of mouse hippocampus.

Neuronal diversity was obvious from the earliest observations of individual neural cells by nineteenth-century microscopists. The great depth of this diversity has only become increasingly obvious, however, with each increment in our anatomical, physiological and molecular toolboxes. The recognition of messenger ligand diversity blossomed throughout the twentieth century and drove recognition of a corresponding receptor diversity (Pert and Snyder, 1973; Hokfelt, 2016; Luo, 2020). The late-twentieth-century advent of molecular genetics then led to recognition of the truly vast scale of receptor diversity, now reckoned at well over a thousand different encoding genes. Now, new single-cell transcriptomic methods are revolutionizing our abilities to grapple neuronal diversity (Zeng and Sanes, 2017; Tasic, 2018; Cembrowski, 2019; Huang and Paul, 2019). Building upon single-cell RNA sequencing (scRNA-seq) data from millions of neurons, transcriptomic neurotaxonomies are offering powerful new frameworks for systematizing neuron diversity and thus predicting their morphologies, connectivity, molecular signaling machinery, and dynamic properties: all factors are obviously critical to neuronal network function. Here we focus upon the new transcriptomic/neurotaxonomic views of modulatory network architectures offered by scRNA-seq methods with an exploration of scRNA-Seq data from mouse hippocampal area CA1.

Two main forms of neuron-to-neuron communication provide the foundation for neuronal network function: (1) fast and anatomically discrete “synaptic” connections, and (2) slower and more spatially diffuse “neuromodulatory” connections that regulate both neuronal membrane excitability and synaptic function. Both synaptic and neuromodulatory signals are highly diverse in messenger ligand identity, receptor selectivity, anatomic architectures, and dynamics. Most synaptic connections depend upon secretion of one of three amino acid neurotransmitters (glutamate, GABA or glycine) or a fourth small molecule, the organic ester acetylcholine, exerting their fast actions directly upon ligand-activated ion channels located just tens of nanometers away across a focal synaptic cleft. Modulatory signaling draws upon a much larger palette of secreted messenger ligands, which includes the very same four small-molecule neurotransmitters but also monoamines such as dopamine, serotonin, norepinephrine, many other small molecules, and the many neuropeptides. These modulatory messengers are sometimes secreted in combination by individual neurons and usually along with one of the fast neurotransmitters (Bucher and Marder, 2013; Hökfelt et al., 2013; Granger et al., 2017; Hokfelt et al., 2018). In contrast to the direct actions of fast synaptic transmitters upon ion channel gating, neuromodulatory messengers act in most cases upon receptors that govern ion channel gating indirectly, *via* molecular cascades that often involve diffusible intracellular messengers and covalent channel modification (Levitan, 1994, 2006; Bucher and Marder, 2013; Levitan and Kaczmarek, 2015; Huang and Zamponi, 2017; Luo, 2020). As both presynaptic and postsynaptic ion channels are foundations of synapse function, modulation of ion channel gating is preeminent among factors that govern the strength and dynamics of synaptic transmission.

Among modulatory receptors, the broadest and most well-studied family are the G protein-coupled receptors (GPCRs, see Box 1). Comparative genomic evidence suggests that the slower GPCR-based forms of signaling recognized today as neuromodulation probably preceded the evolutionary inventions of neurons and synapses (de Mendoza et al., 2014; Arendt, 2021; Jekely, 2021). Ancestral, very small animals probably coordinated their multiple cell types and generated their slow but (back then) perfectly competitive behaviors by slow GPCR-based forms of cell-cell signaling resembling today’s modulatory signaling. Evolutionary pressures that placed a premium on an animal’s size, speed, and ability to learn then probably drove evolution of the extended arborized forms of neurons and the focal nature of fast synaptic transmission (Arendt, 2020; Jekely, 2021). The very large numbers of ancestral GPCR genes expressed in all of today’s higher animals (de Mendoza et al., 2014) suggests that the “ancient” forms of slow signaling remain essential as contributors to the fine-tuning and adaptability of the “newer” synaptic networks.

**Box 1. Primer on GPCR control of ion channels and synapses.**

G protein-coupled receptors play central roles in the homeostasis and modulation of neuronal network function (Gainetdinov et al., 2004). A large part of how they do so is through powerful regulation of ion channels and thus of membrane excitability (Levitan, 1994) and synaptic transmission (Brown and Sihra, 2008). Though GPCRs comprise a very large family of membrane receptors, enormously diverse in their ligand selectivities, they share many basic biochemical principles of operation (Rosenbaum et al., 2009; Hilger et al., 2018). We’ll focus in this primer on GPCR signaling mediated through receptor coupling to ion channels *via* heterotrimeric G proteins.

**(A)** GPCR-G protein activation. Binding of an activating ligand (agonist) to a GPCR promotes or stabilizes an active receptor conformation, which is allosterically “coupled” to the engagement of specific heterotrimeric G protein(s) (Zachariou et al., 2012; Weis and Kobilka, 2018). Heterotrimeric G proteins are composed of an α-subunit, which binds guanine nucleotide and largely determines selectivity for coupling with GPCRs, and β and γ subunits which form a stable βγ subcomplex. GPCR coupling promotes dissociation of GDP from the α subunit followed by binding of GTP. This activates the α subunit and “undocks” the βγ subcomplex (Mahoney and Sunahara, 2016).

**(B)** Downstream effector control by G proteins. The GTP-bound G protein α-subunit transduces signaling by regulating enzymes. Heterotrimeric G proteins are traditionally classified into three major classes–Gq, Gs, and Gi/o–based on the selectivity of their α-subunits for downstream enzyme control (Zachariou et al., 2012). Activated (GTP-bound) Gq-class α-subunits stimulate phospholipase C enzymes, Gs-class α-subunits stimulate adenylyl cyclase enzymes, and Gi/o-class α-subunits inhibit adenylyl cyclase enzymes. Phospholipase C catalyzes conversion of the membrane phospholipid PIP2 to the membrane lipid diacylglycerol (DAG) and soluble inositol trisphosphate (IP3), both of which act as intracellular “second messengers.” Adenylyl cyclase catalyzes conversion of ATP to cyclic AMP, which also acts as a second messenger. In addition, Gi/o-class heterotrimeric G proteins are major sources of undocked βγ subunits; Gq and Gs can also produce βγ subunits but generally do so in smaller amounts (Touhara and MacKinnon, 2018).

**(C)** Second messenger actions. Both DAG and cyclic AMP stimulate protein kinases that phosphorylate and thereby regulate many (and possibly all) types of ion channels and synaptic proteins. IP3 binds to receptors that amplify the dynamics of intracellular ionic calcium, another potent intracellular messenger that can stimulate protein kinases to impact channels and synapses.

**(D)** Membrane phospholipid signaling. Stimulation of phospholipase C by Gq-class α-subunits can cause significant changes in membrane phospholipid composition, such as depletion of phosphatidylinositol 4,5-bisphosphate (PIP2). This impacts the function of various membrane proteins, including channels and synaptic effectors directly, independent of other second messenger effects (Hille et al., 2015).

**(E)** Gβγ signaling. Undocked G protein βγ subcomplexes can exert direct actions upon ion channels, independent of enzyme regulation by α-subunits or the production of second messengers (Herlitze et al., 1996; Smrcka and Fisher, 2019). Two direct actions of βγ subunits that are particularly important to neuromodulation are activation of inwardly rectifying potassium channels and inhibition of voltage-gated calcium channels.

**(F)** Kinetics of cascade activation and deactivation. GPCR impact upon ion channels and synaptic protein signal transduction processes play out over diverse time courses, ranging from a fraction of a second (direct βγ-to-channel) to many minutes where the dynamics of second messenger production, enzymatic cascades and protein phosphorylation-dephosphorylation are involved. GPCR activity can be diminished within seconds through receptor phosphorylation and binding of arrestin proteins (Ahn et al., 2020). G proteins are deactivated by hydrolysis of the bound GTP to GDP by an enzymatic activity that is intrinsic to the α-subunit; this deactivation rate can range from several seconds to less than a second, depending on binding to the α-subunit of “regulator of G protein signaling” (RGS) proteins that accelerate hydrolysis (Masuho et al., 2020).

**(G)** Additional mechanisms of GPCR signaling. We focus in the present discussion on signaling mediated by classical coupling between GPCRs to heterotrimeric G proteins. This transduction mechanism is sufficient to explain many GPCR-elicited signaling effects, but we note that additional transduction mechanisms may also contribute to physiological neuromodulation by GPCRs. These include signaling mediated independent of G proteins from GPCR-arrestin complexes, signaling by an alternate GPCR-G protein complex that also contains arrestin, and signaling through the direct interaction of GPCRs with ion channels (Zamponi, 2015; Sutkeviciute and Vilardaga, 2020).

In the present writing, we explore new transcriptomic neurotaxonomy perspectives on neuromodulatory signaling architectures, using rodent hippocampus as an illustrative and particularly well-studied case in point (Cembrowski et al., 2016a,b; Cembrowski and Menon, 2018; Cembrowski, 2019; Cembrowski and Spruston, 2019). Our treatment will center upon GPCR transcriptomes but will also touch upon transcriptomes of ion channels (the primary downstream targets of GPCR activation), signal transducing G proteins, and genes encoding precursors to neuropeptides (the largest and most diverse family of brain GPCR ligands). A more thorough treatment of neuromodulation in hippocampus or elsewhere would consider many other classes of receptors (e.g., receptor tyrosine kinases), other classes of ligands (e.g., neurotrophins and other cytokines), other effector targets (e.g., synaptic proteins other than ion channels and regulators of gene expression) and many other intracellular signaling molecules (e.g., kinases, phospholipases). We include neurotaxonomic type-mean signature data for some of these other modulators as Supplementary Material, but we leave their due exploration and discussion for another day.

We’ll not attempt an expert’s review of new transcriptomic or neurotaxonomic methods. Rather, we’ll focus on a few key observations that emerge from examination of a large dataset published recently with a corresponding neurotaxonomy (Yao et al., 2021) and highlight findings we believe are likely to generalize. We do so through a series of vignettes, admitting no attempt at completeness. We encourage the interested reader to continue the journey and provide links to data and code that may help the interested reader explore this or similar datasets more deeply and broadly.

## Materials and Methods

We focus here on transcriptomic expression patterns of genes encoding proteins likely to play key roles in neuromodulatory signaling in area CA1 of mouse hippocampus. Our candidate neuromodulators include GPCRs, heterotrimeric G proteins, ion channel subunits, and neuropeptide precursor proteins (NPPs). We draw solely upon RNA-Seq expression datasets and a neurotaxonomy described in recently published work (Yao et al., 2021) and available for download and interactive exploration at https://portal.brain-map.org/atlases-and-data/rnaseq. Methods of data collection and development of the deep hierarchical neurotaxonomy based on profiling ∼1.3 million cells and comprising 388 neuron types across the entirety of isocortex and hippocampal formation are described fully in the cited resource paper (henceforth, “Yao21” for short). Of that work’s 388 types, 124 neuron types represent cells sampled from the hippocampal formation. For the present analysis we rely upon a subset of the data (3,305 cells) obtained by the deepest of the Yao21 scRNA-seq methods (SMART-Seq v4) from cells of hippocampal region CA1. The present analysis is restricted to a 42-type subset of the 388-type Yao21 taxonomy, comprising the 29 GABAergic and 13 glutamatergic neuron types schematized in Table 1. These 42 types were selected from the 124 types found across the entire hippocampal formation based on a requirement that the Yao21 SMART-Seq dataset include at least 16 cells sampled from hippocampal area CA1. Further particulars of our sifting down to this robustly expressed 42-neuron-type CA1 taxonomy are tabulated in Supplementary Materials.

**TABLE 1:** A hierarchical neurotaxonomy comprising 2 classes, 6 subclasses, 14 supertypes, and 42 types of neurons found in area CA1 of mouse hippocampus.

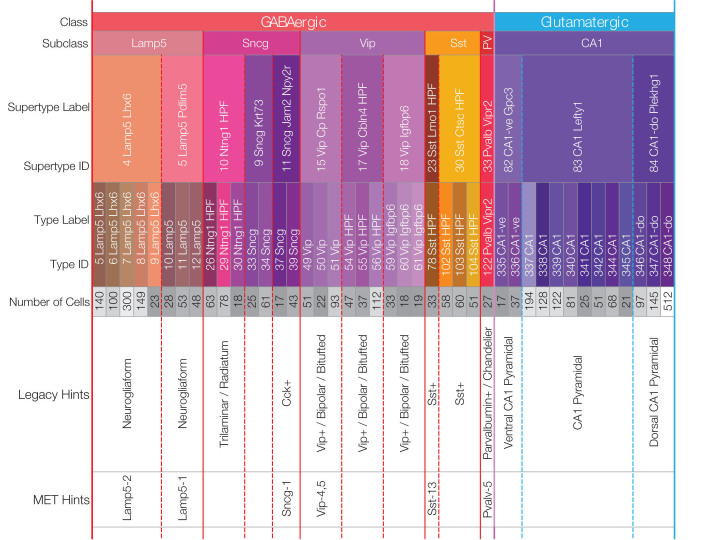

*This taxonomy provides a framework for the present analysis: we have extracted just elements relevant to hippocampal area CA1 from the much larger (388-type) mouse cortex taxonomy developed by Yao et al. (2021) (see also Section “Materials and Methods”).*

*The table indicates the number of neurons of each type for which SMART-seq v4 transcriptomes were available: only types represented by more than 16 cells are included.*

*A correspondence of the 14 transcriptomic supertypes tabulated here to traditional anatomic or immunohistochemical “legacy” or “MET” (Gouwens et al., 2019, 2020) neuron types is suggested tentatively.*

*Subsequent figures will designate the 42 CA1 neuron types using a compressed version of the type-hierarchy color mosaic introduced here.*

To launch the analysis presented here, we compiled an initial broad list of 1,749 candidate genes that we consider (somewhat arbitrarily) “modulation-related.” Of these, we found that messenger RNAs corresponding to 1,111 genes are represented in the Yao21 SMART-Seq dataset at mean levels greater than 10 CPM (far above measurement “noise”) in at least one of the 42 CA1 neuron types. Area CA1 expression data for all 1,111 genes are tabulated in both graphical and numeric forms in our Supplementary Materials. Again somewhat arbitrarily, we selected a more focused subset of 595 genes we deemed likely to be of greatest interest in fathoming hippocampal neuromodulation. The 595 comprise genes encoding 151 GPCRs, 55 proteins involved directly in GPCR signal transduction, 178 ion channels, 36 NPPs, and 175 other signaling proteins. Here we describe neuron-type specific expression of key subsets of these genes chosen to lay outlines of a network-level view of hippocampal neuromodulation, emphasizing the possible importance of neuron-type-specificity in vectorial signaling between functionally distinct neuronal subpopulations.

**FIGURE 1:**
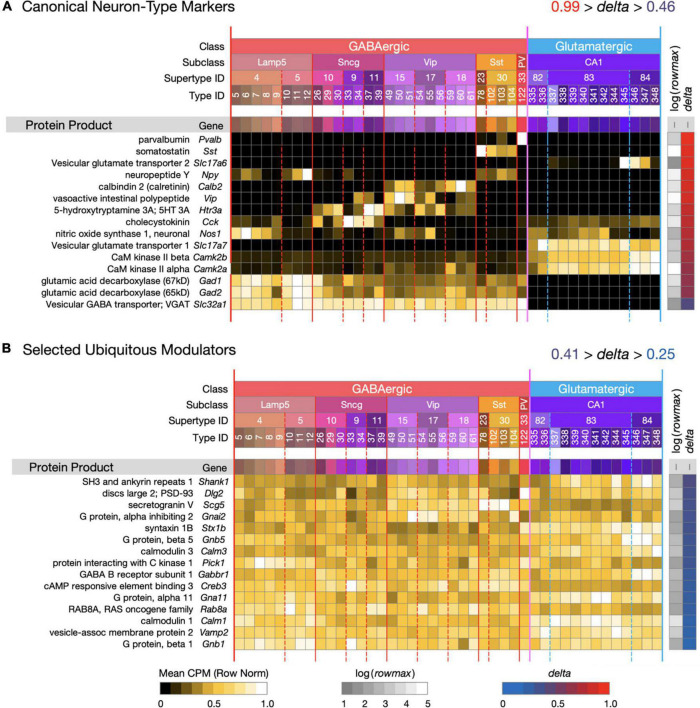
Introduction to a neurotaxonomic gene expression matrix display format. (A) Signatures of 15 genes encoding proteins commonly referenced as molecular markers of neuron type. (B) Signatures of 15 modulatory genes expressed much more ubiquitously. These maps are based on (gene) × (type) matrices representing row-normalized type-mean CPM values according to the “Mean CPM” color scale at bottom. Each row is flanked by a protein and gene label at left and at right by values representing log(rowmax) and delta (see Eq. 1) encoded according to the color scales at bottom. Columns are denoted by a compressed version of the Table 1 taxonomy color mosaic and by colored vertical lines extending from the mosaic through the expression matrices. The signatures of individual genes are ordered here and in all subsequent figures in descending delta order. Subsequent figures will represent expression signature results using these very same graphic conventions. For this and all subsequent expression matrix displays, numerical versions are available as downloadable Supplementary Material.

**FIGURE 2:**
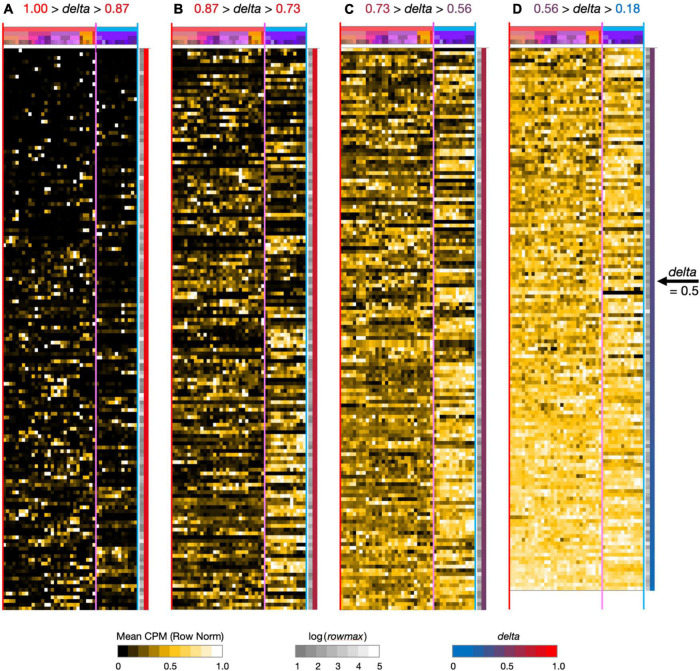
Neurotaxonomic type-mean signatures for 595 modulator genes expressed at high levels (at least one type-mean CPM > 10) in hippocampal area CA1. Note that in successive panels (A–B) genes are ordered by continuously descending values of delta, as is evident from the continuous red-to-blue color gradient extending along the right-hand panel margins. Matrix labels are suppressed here to avoid nil legibility, but a fully annotated and legible version is downloadable as Supplementary Material.

**FIGURE 3:**
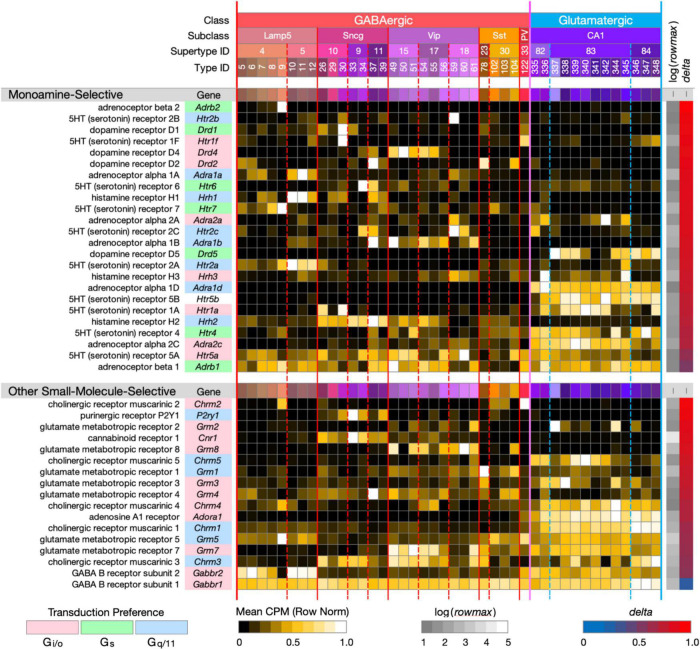
Neurotaxonomic type-mean signatures for 41 genes encoding small-molecule-selective GPCRs. (Upper panel): genes that encode 24 GPCRs selective for the monoamine neuromodulators norepinephrine, dopamine, serotonin, and histamine. (Lower panel): genes that encode 17 GPCRs selective for the additional small-molecule modulators endocannabinoids, adenosine, ATP, and the synaptic neurotransmitters GABA, glutamate, and ACh (each displayed according to the format introduced in Figure 1). With one exception, these GPCR genes all exhibit very high type specificity (mean delta = 0.85 for the monoamines; mean delta = 0.78 for the others). The one exception is Gabbr1 (delta = 0.34).

**FIGURE 4:**
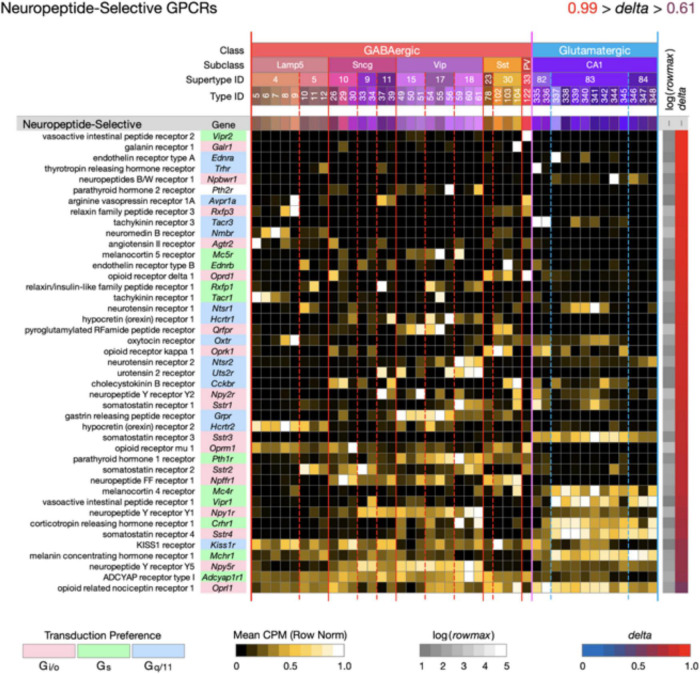
Neurotaxonomic type-mean signatures for 43 genes that encode neuropeptide-selective GPCRs (NP-GPCRs). All of these GPCR genes exhibit very high type specificity (mean delta = 0.87).

**FIGURE 5:**
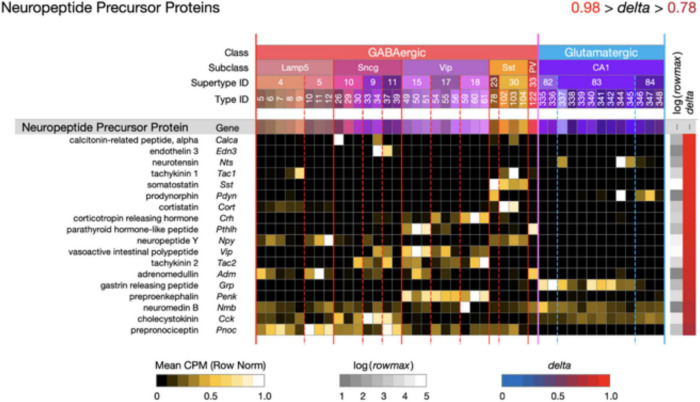
Neurotaxonomic type-mean signatures for 18 genes that encode neuropeptide precursor proteins (NPPs). All of these GPCR genes exhibit extremely high type specificity (mean delta = 0.92) and all encode peptides cognate to at least one of the NP-GPCR genes profiled in Figure 4.

**FIGURE 6:**
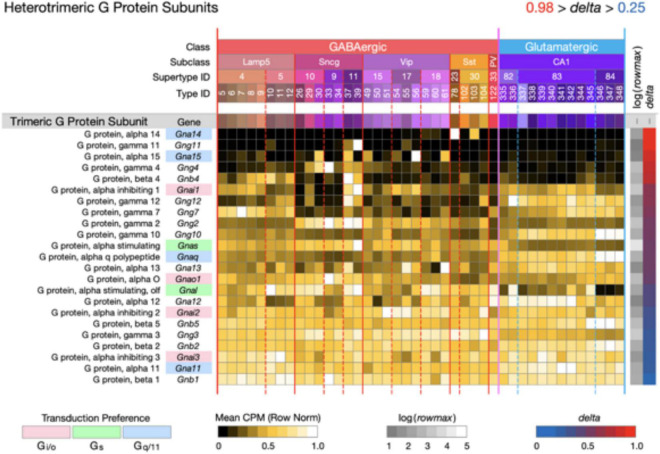
Neurotaxonomic type-mean signatures for 23 genes that encode heterotrimeric G protein subunits. Expression of most of these subunits in CA1 is very much less type-specific (mean delta = 0.58) than that of the CA1 GPCRs and NPPs. The alpha subunit genes that confer GPCR preference are indicated here using the same gene symbol and color shading scheme as used in Figures 3–5.

**FIGURE 7:**
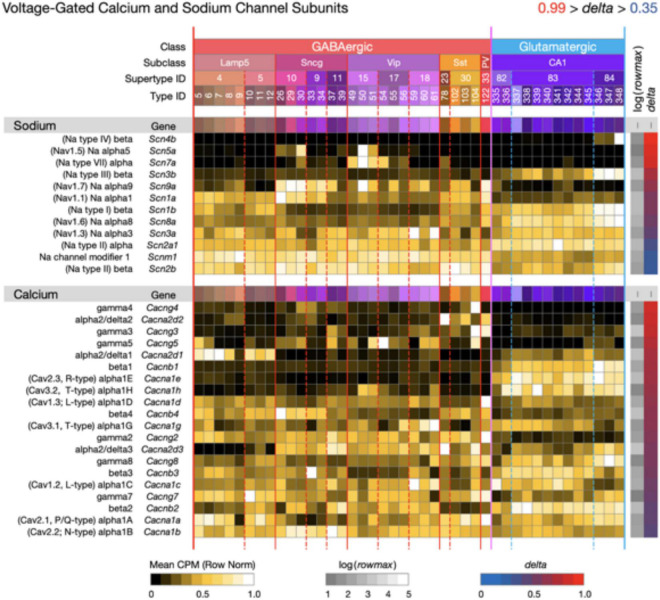
Neurotaxonomic type-mean signatures for voltage-dependent sodium and calcium channel subunits. Upper panel: 12 genes that encode voltage-dependent sodium channel subunits. Lower panel: 20 genes that encode voltage-dependent calcium channel subunits (lower panel). Both gene sets exhibit wide ranges of type-specificity and accordingly modest mean delta values (Sodium mean delta = 0.65; Calcium mean delta = 0.68).

**FIGURE 8:**
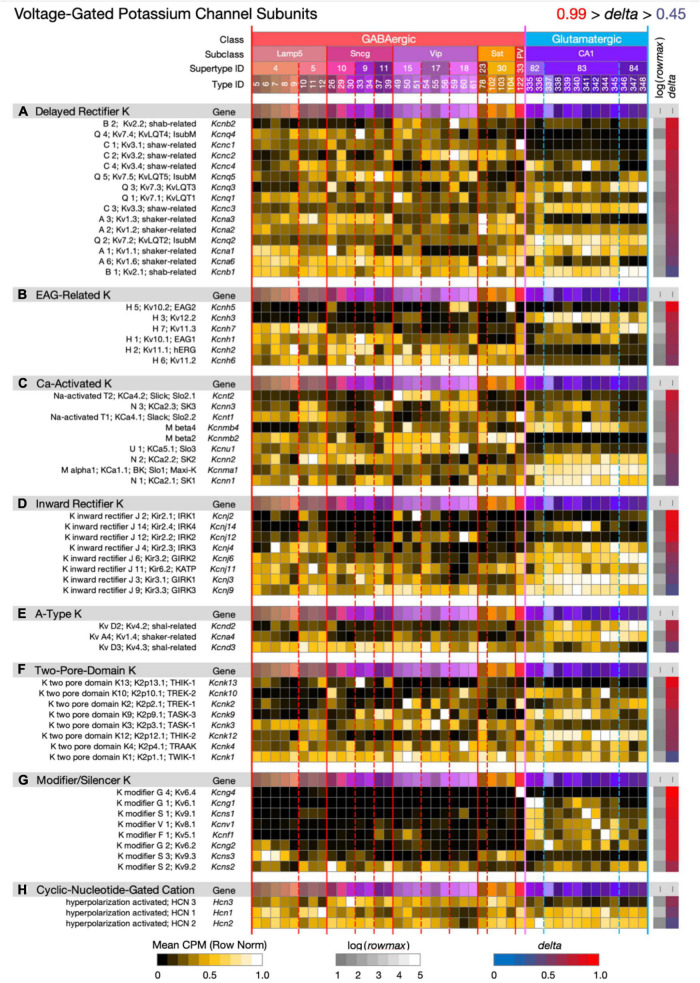
Neurotaxonomic type-mean signatures for 60 genes that encode voltage-dependent potassium channel subunits, eight categories. Most of the eight gene sets exhibit wide ranges of specificity and accordingly modest mean delta values: (A) 0.69, (B) 0.72, (C) 0.67, (D) 0.70, (E) 0.66, (F) 0.70, (G) 0.87, (H) 0.56. The modifier/silencer genes are interesting as consistently high-delta exceptions.

**FIGURE 9:**
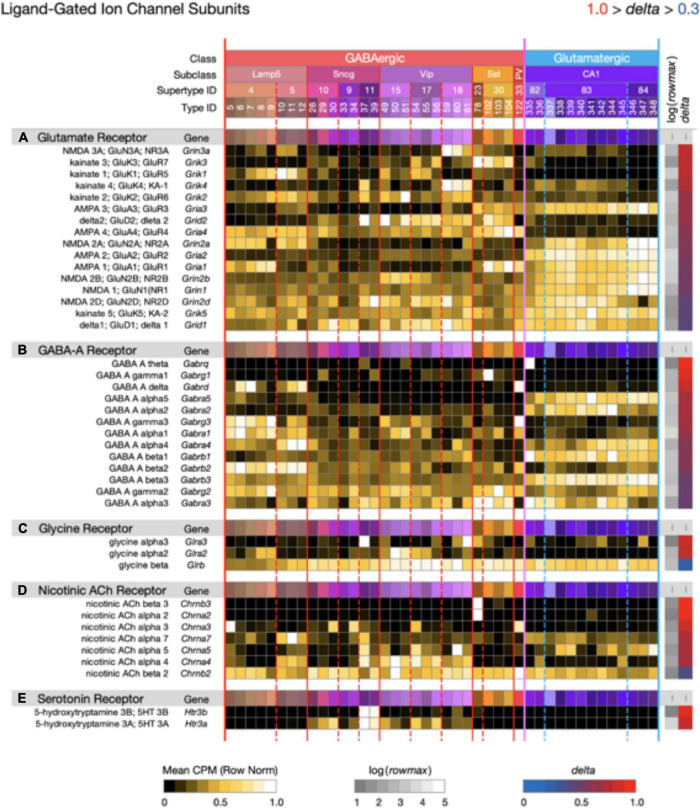
Neurotaxonomic type-mean signatures for 41 genes that encode ligand-gated channel subunits, divided into five categories based on principal endogenous agonist. Three of the five gene sets exhibit wide ranges of specificity and accordingly modest mean delta values: (A) 0.67, (B) 0.72, (C) 0.68, (D) 0.80, (E) 0.91. The consistently higher delta values of ACh and serotonin receptor signatures are intriguing in view of their noteworthy modulatory, as opposed to strictly synaptic, roles.

**FIGURE 10:**
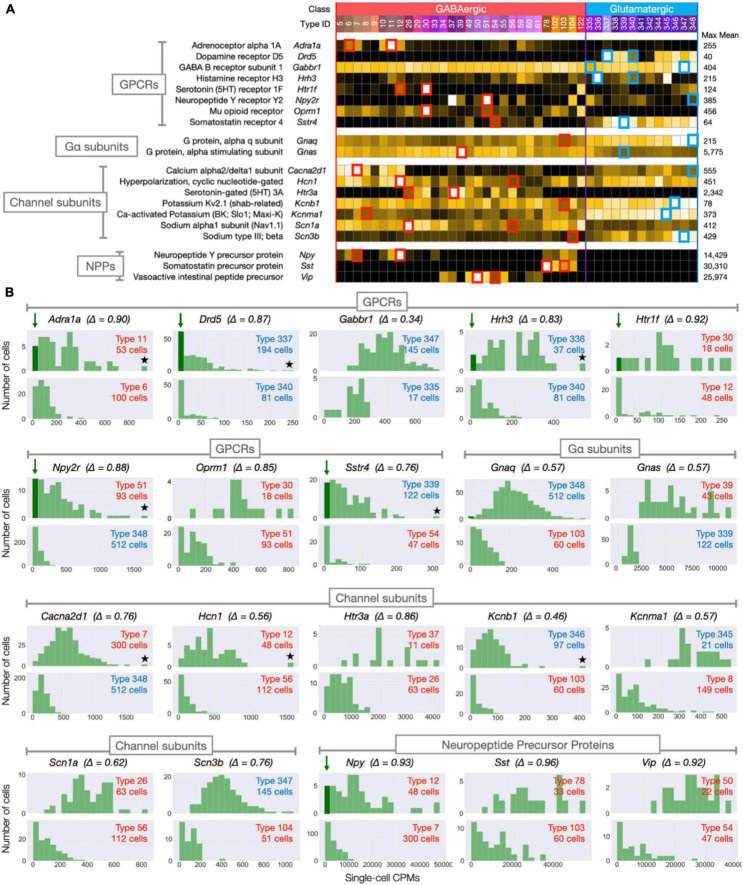
Within-type transcript count distributions for selected (gene) × (type) subsets. (A) Representative type-mean signatures (drawn from Figures 3–9) for 8 GPCRs, 2 G-protein subunits, 7 channel subunits, and 3 neuropeptide precursor proteins. Small red (GABA types) and blue (Glut types) squares highlight gene × type subset means selected for single-cell histograms in panel B. One square per gene row highlights highest mean CPM type, a second highlights another type with a mid-range mean CPM value (∼25% the maximum mean). (B) Histograms representing distributions of single-cell CPM values within highlighted gene × type subsets (Δ indicates gene’s CA1 delta value). Top panel for each gene represents the max-mean type; bottom panels the mid-range type. Arrows and darkened vertical bars in seven panels indicate distributions suggesting anomalously zero or low CPMs value; stars in five panels indicate evident high-CPM outliers (see text).

We developed the (gene) × (neuron type) mean expression matrices represented in all data figures below by distilling Yao21 SMART-Seq (cell) × (gene) matrices representing 73,363 single cells and 45,769 mapped genes (19,751 protein-coding), summing exon and intron reads. These data matrices are annotated by the Yao21 metadata tables as to the brain region from which each cell was sampled and the transcriptomic cell type cluster to which each was assigned. These metadata tables allowed assignment of 3,305 single-cell samples to hippocampal area CA1 and each sample to one of the 42 type clusters represented in Table 1. Figures 1–10 below represent transcript abundance estimates (normalized as counts per million mapped reads, CPMs), as mean values for each indicated gene within each type cluster. We refer in the following to the set of means across all 42 type clusters for any given gene as that gene’s “type-mean expression signature” (often shortened to “expression signature” or simply “signature”).

To quantify the neuron-type-specificity reflected in expression signatures on a gene-by-gene basis, we define a metric *delta* for each gene as follows:


(1) $$delta=\frac{n-\sum_{i=1}^{n}\mu_{i}/\mu_{\max}}{\left(n-1\right)}$$

where *μ_*i*_* is mean CPM within each type *i* and *μ_*max*_* is the maximum mean value across all *n* types (*n* = 42 in this case). Possible *delta* values range from 0 (non-type-specific: all type means equal) to 1.0 (maximally type-specific: all type means but one = 0, with non-zero expression in just one type).

Several caveats should be mentioned. (1) While our analysis is based on one of the deepest and most quantitative scRNA-seq methods presently available, the data is still subject to known biases and stochastic sampling limitations. The bias of likely greatest concern for the present work lies in that only cell somata were sampled, while many important neuronal transcripts are known to be localized preferentially to dendrites or axons (Glock et al., 2021) and may thus be greatly underestimated. (2) While alternate splicing of mRNA is known to dramatically transform the functional properties of many protein products, the dataset we have drawn upon does not discriminate amongst splice variants. (3) Many of the GABAergic neurons profiled for the present study were harvested from hippocampal areas other than CA1, as justified by findings that GABA cell transcriptomes are generally conserved across areas (Tasic et al., 2018; Yao et al., 2021). (4) Several type clusters represented in area CA1 were excluded from consideration due to low numbers of cells (<16) per cluster. (5) Finally, it should be emphasized that numbers of cells per type category reported here and in the Yao21 resource publication and dataset do not correspond faithfully to actual relative abundance of neurons in the source tissues. This is a consequence of both engineered sampling biases and biases resulting from type-dependent differential recovery during cell soma collection (Yao et al., 2021).

The scripts and worksheets used to generate all data figures below from the primary Yao21 resources are provided here as Supplementary Materials and provide access to all displayed data (and much more) in numeric form. Supplementary Materials also offers evidence that the type-mean expression signatures displayed in Figures 1–10 are statistically robust by resistance to subsampling.

## Results

Table 1 represents the 42-type hippocampal CA1 taxonomy we sifted from a much larger, cortex-wide taxonomy recently published by Yao et al. (2021) (see Section “Materials and Methods”). The 42 types are partitioned here according to higher-level categories of the hierarchical Yao21 taxonomy (classes, subclasses, and supertypes). For all that follows, we relied upon assignments of each of the 3,305 single-cell CA1 samples to one of these 42 types by the Yao21 resource. The table also includes hints at likely correspondences between the Yao21 taxa and other past and present neuron classification schemes.

Figure 1 introduces the matrix display format we’ll use to represent type-mean expression signatures derived from the curated CA1-focused dataset. Mean CPM values for each gene (rows) and each neuron type (columns) are encoded by the indicated color keys and displayed in row-normalized matrix form. The 42 CA1 neuron types are keyed by the taxonomic color mosaic introduced in Table 1. Figure 1 shows two sets of 15 genes representative of relatively high (1A) and relatively low (1B) cell type-specificity, as defined by calculated *delta* values (see Section “Materials and Methods”) and encoded by the *delta* color key. The 15 high-*delta* gene expression signatures in Figure 1A clearly exhibit the strongly differential expression patterning that has led to historical use of proteins encoded by these 15 genes as molecular markers of cell type (e.g., see Tremblay et al., 2016). Signatures of the 15 low-*delta* genes represented in Figure 1B, on the other hand, exhibit the relatively constant, type-independent expression patterns expected from genes whose protein products are generally thought to be ubiquitous as synaptic or intracellular signaling proteins. Within each panel, genes are sorted to display those with the highest cell type-specificity of expression (*delta* approaching 1) at the top and those with the lowest cell type-specificity of expression (*delta* approaching 0) at the bottom. We follow the same convention in subsequent Figures 2–9.

To see what such visualizations might tell us about hippocampal neuromodulation, we began with a broad survey of all 1,749 of our candidate modulation-related genes and found that 595 were expressed at a high level (type-mean > 10 CPM) in at least one of the 42 CA1 types. Figure 2 provides an overview of expression signatures of these 595 genes. Display here is similar to that introduced by Figure 1, except that display of identifying gene symbols and taxonomic labels is suppressed here due to graphics constraints (Fully annotated and numerical versions of the full 1,749- and 595-gene expression matrices can be found in Supplementary Material). The entire set is sorted by descending *delta* values and displayed in four contiguous segments from *delta* = 1.00 at the top of panel A to *delta* = 0.18 at the bottom of column D.

Perhaps the simplest conclusion one can draw from the Figure 2 overview of 595 modulator genes is that the great majority are expressed in highly type-specific fashions. Type specificity is clear from visual inspection of columns (A–C) and the ordering *delta* values stay well above 0.5 until halfway down column (D): thus, over 83% of the 595 genes thus exhibit strong type specificity and correspondingly *delta* values, >0.5. In addition, inspection of the highest-*delta* column (A) shows that every one of the 42 types is a “hot-spot” of expression for at least one gene, even in this sparsely filled regime. Note also that even for genes where expression is detected in relatively large fractions of the 42 neuron types (columns B–D, *delta* values below 0.87), clear distinctions between GABAergic and glutamatergic types (demarcated by vertical magenta boundary lines) are quite apparent in the gestalt.

We have selected 258 of the 595 genes represented in Figure 2 for further exploration here, according to our judgment that certain GPCRs, G proteins, ion channels, and NPPs are likely to have the greatest presently interpretable relevance to CA1 neuromodulation. Figures 3–9 display type-mean matrices depicting expression signatures of these 258 genes grouped in nine major categories: GPCRs selective for monoamine-selective, other small-molecules and neuropeptides, NPPs, G protein subunits, subunits of ion channels selective for sodium, calcium, and potassium and ligand-gated ion channels (Similar matrix display visualizations of all 595 modulator genes expressed at high levels in CA1 hippocampus, along with a still more comprehensive set of 1,111 modulator genes expressed at any significant level in CA1, can be found in Supplementary Materials).

Figure 3 (upper panel) displays strikingly type-specific (mean *delta* = 0.85) expression signatures for 24 genes encoding GPCRs selective for norepinephrine, dopamine, histamine and serotonin, modulatory agonists deeply implicated in research on mechanisms of learning, human neuropsychiatric disorders and related therapeutics. These modulators are secreted at varicosities along axons ramifying extensively from hindbrain, midbrain and hypothalamic nuclei into the hippocampus and many other forebrain regions. These GPCRs represent three major classes of G protein coupling preference, G_*i/o*_, G_*s*_, and G_*q/11*_, as indicated by the “Transduction Preference” color key.

Figure 3 (lower panel) displays highly type-specific (mean *delta* = 0.78) signatures for 17 GPCRs selective for additional small-molecule modulators endocannabinoids, adenosine, ATP and the synaptic neurotransmitters GABA, glutamate, and ACh. These modulators may be locally released or of remote axonal origin. The synaptic transmitters act *via* these GPCRs in slower, modulatory roles distinct from those of the ligand-gated receptor/channels (characterized below) that support fast synaptic transmission. Interestingly, these GPCRs represent only two classes of coupling preference, G_*i/o*_ and G_*q/11*_.

Figure 4 displays highly type-specific (mean *delta* = 0.87) expression signatures for 43 neuropeptide-selective GPCRs (NP-GPCRs). The endogenous agonists for these receptors are the neuropeptides, some secreted locally by hippocampal neurons (see below), and others reaching the hippocampus *via* axons projecting from hypothalamus and other distant brain regions. Exogenous ligands of special interest include the entire opioid pharmacopeia and many other small molecules or synthetic peptides in use or under investigation for therapeutic purposes (Muttenthaler et al., 2021).

Two general lessons emerge from Figures 3, 4. First, numerous GPCR genes are expressed in each and every neuron type (A later section will quantify this conclusion at the level of single cells). Second, most of the 84 GPCR genes analyzed here are expressed in a very highly type-dependent patterns. In some cases, patterning appears to reflect mainly class, subclass or supertype categories, but more commonly patterning is evident down to the single-type level.

Figure 5 displays extremely type-specific (mean *delta* = 0.92) expression signatures for 18 genes that encode NPPs. Transcripts of one or more NPP genes are among the very most abundant in almost all individual hippocampal neurons, and the neuropeptide products resulting from NPP proteolysis constitute the largest and most diverse family of neuromodulatory ligands. It is noteworthy that genes encoding GPCRs cognate to neuropeptides encoded by each of the 18 NPP genes listed in Figure 5 are expressed in hippocampus, as indicated in Figure 4. Such cognate pairing suggests that hippocampus may harbor dense peptidergic modulatory networks, as have been suggested in other brain regions and species (Smith et al., 2019, 2020; Smith, 2021).

Figure 6 displays expression signatures for 23 heterotrimeric G protein subunits. As discussed in Box 1, these subunits compose the most common and well-studied transducers of GPCR activation. The alpha subunit differences that confer GPCR preference are indicated here using the same gene symbol color shading scheme as used in Figures 3–5. Expression of most of these subunits in CA1 is notably much less type-specific (mean *delta* = 0.58) than that of the CA1 GPCRs and NPPs. It may be, therefore, that GPCRs quite diverse in their ligand selectivity converge to a much less diverse set of signals within the neuron. Any such inferences may be subject to change, however, as we learn more about sub-cellular localization of both GPCRs and their ion channel targets, and about the diffusion dynamics of intracellular second messengers.

Figures 7–9 display expression signatures for genes encoding 97 voltage-gated and 41 ligand-gated ion channel subunit proteins. Display as three separate figures was necessitated by the large number of channel subunit genes expressed in CA1, each with strong and distinctive type specificity, localization, and probable functional impacts. The protein product labels in these figures make feeble attempts to capture some alignment between subunit gene symbols and channel terminologies that have arisen during many decades of intense interest in ion channel physiology and molecular biology. More complete discussions of these alignments can be found elsewhere (Hille, 2001; Levitan and Kaczmarek, 2015; Luo, 2020; Alexander et al., 2021).

Almost all gated ion channels are composed of multiple subunits encoded by different genes, with subunit co-assembly tendencies often indicated by alpha, beta, gamma gene symbol designations. Major functional properties of the resulting multi-subunit channel can be influenced by all components and subunits can assemble in widely varied combinations. The result is a possible combinatorial explosion in the major functional channel properties such as gating and permeability that establish distinctive characteristics of neuronal excitability and the bidirectional linkage of membrane potential dynamics to synaptic function.

Figure 7 displays expression signatures for 12 sodium (upper panel) and 20 calcium (lower panel) channel subunit genes. Voltage-dependent sodium channels are essential to membrane excitability (i.e., action potential firing, a.k.a. “spiking”) in almost all neurons, although voltage-dependent calcium channels may be evolutionary precursors in this capacity and may remain predominant in some cases. Voltage-dependent calcium channels, typically opened in response to a sodium spike, are absolutely essential to almost all secretion of both synaptic transmitters and modulatory ligands. Both sodium and calcium channels are major targets of modulatory signaling (Levitan, 2006; Levitan and Kaczmarek, 2015; Huang and Zamponi, 2017) (e.g., *via* protein phosphorylation and other downstream impacts of GPCR activation) and such modulation therefore may impact both membrane excitability and the strength and dynamics of synaptic transmission in profound ways.

Figure 8 displays expression signatures for 60 voltage-dependent potassium channel superfamily genes in eight categories with terminologies that reflect a long history of physiological and molecular discovery. The many genes and categories are nonetheless also a true reflection of the depth and breadth of variations in potassium channel structure and function. Potassium channels account for the action potential downstroke, as well as being principal determinants of critical subthreshold membrane behaviors such as spike-frequency encoding. In both of these roles, potassium channels loom as major factors governing synaptic strength and dynamics. Potassium channels are also major determinants of the complex, non-linear electrotonus of dendritic arbors, which is increasingly recognized as a major element in memory formation and neuronal computation. Finally, some members of this superfamily, the Ca-activated K channels and cyclic-nucleotide-gated (HCN) cation channels (the latter being less selective for potassium over other cations) are gated by intracellular calcium ions or the cyclic nucleotides, cGMP and cAMP. Since potassium channels are also major targets of GPCR-based modulation, they must be reckoned as central factors in all adaptive neuronal network function.

Figure 9 displays expression signatures for 41 ligand-gated ion channel genes in five categories denominated by identities of the principle endogenous agonist, three amino acids and two small molecule enzyme products, acetylcholine, and serotonin. Channels assembled from these subunits are gated directly by the agonist and are therefore well suited to fast postsynaptic potential generation. They might thus be thought of primarily as targets, rather than mediators of slow neuromodulatory signaling. This distinction is imperfect and potentially misleading, however, especially for the ACh- and serotonin-gated channels, which are often cast in modulatory as well as strictly synaptic roles (Govind et al., 2012; Arroyo et al., 2014; Sizemore et al., 2020).

Figures 3–9 displayed 258 signatures representing neuron-type-specific expression of 258 genes encoding GPCRs, G-protein subunits, ion channel subunits, and NPPs. To generate each signature, many single-cell CPM values for the given gene were aggregated as one mean CPM value per neuron type. To offer a compact glimpse of single-cell CPM variations within types, prior to such aggregation, we selected 20 genes and two neuron types per gene as representative. Figure 10A reproduces type-mean signatures for that set of 20 genes (encoding 8 GPCRs, 2 G-protein subunits, 7 channel subunits, and 3 NPPs). Figure 10A also highlights the two types chosen for each gene: one type showing highest mean expression and a second chosen to represent mid-range expression (approximating 25% of that peak mean). Figure 10B shows a pair of histograms for each of the 20 genes, representing distributions of CPM values for both high- and mid-expressing types. Arrows and darkened zero-rank bars in Figure 10B highlight seven histograms suggestive of anomalously low or zero expression in the peak mean cell types. Interestingly, genes encoding cell-cell signaling molecules (six of the eight GPCRs and one of three NPPs) account for all these possible anomalies. Stars in seven Figure 10B panels highlight evident high-CPM outliers. It may be relevant that five of these seven high-end outliers happen to mark the same genes marked for low-end anomalies (Supplementary Figure 1 offers an alternative visualization of outlier samples). It is possible that technical factors in sequencing or classification might explain these outliers: it is common for RNA-Seq data to be summarized by aggregation as trimmed means to eliminate outlier impacts. We have avoided such trimming here, however, as an unnecessary complication given the relatively minimal outlier occurrence evident in Figure 10B.

**FIGURE 11:**
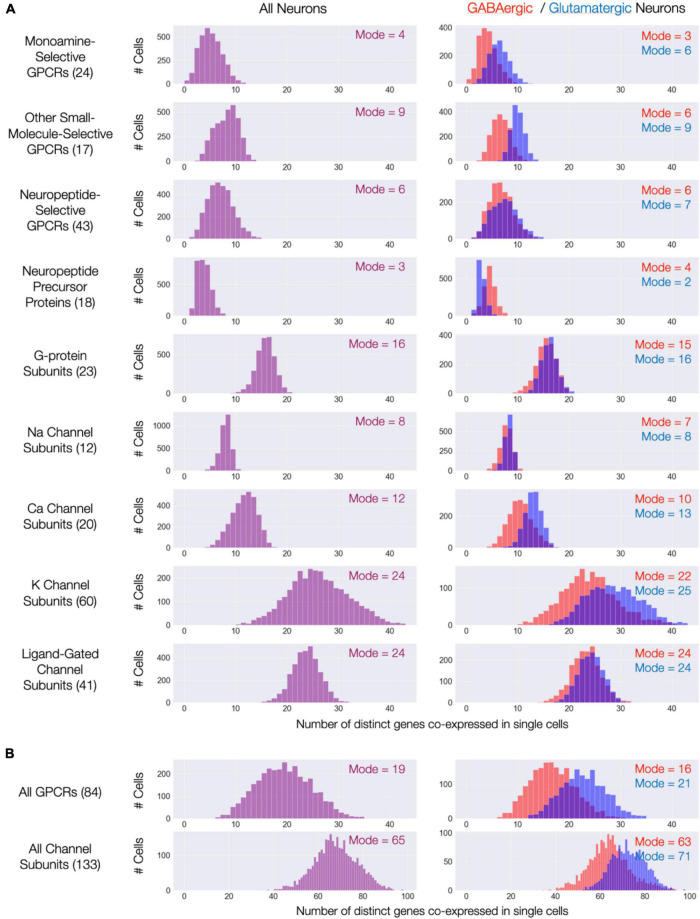
Single-cell co-expression of modulatory genes from the nine Figures 3–9 gene sets. (A) Histograms of numbers of distinct genes co-expressed at high levels (each > 10 CPM) from the sets named (gene set size in parentheses). Left: distributions across all 3,305 CA1 neurons; Right: separate distributions for 1,807 GABAergic (red) and 1,498 Glutamatergic neurons (blue). Modes of each distribution indicated numerically within each display panel. (B) Similar representations aggregating all 84 GPCR genes and all 133 ion channel genes.

The expression maps of Figures 3–9 show very substantial type-specific co-expression of many genes within each of nine broad categories defined by those maps. To avoid possible misinterpretation of these aggregated data, we have tallied multi-gene co-expression at the level of individual neurons. Figure 11A displays the results as three histograms for each of the nine gene categories, with the left column representing all 3,305 individual CA1 neurons and the right column displaying results separately for the GABAergic and glutamatergic neurons. The right columns in Figure 11A indicate that co-expression patterns for most gene categories differ somewhat between GABAergic and glutamatergic neurons: in most cases, a higher degree of co-expression is apparent for glutamatergic neurons while, on the other hand, NPP co-expressions appears substantially greater for GABAergic neurons. Nevertheless, the vast majority of neurons in area CA1–whether inhibitory or excitatory–express at least one NPP transcript and often more than one. Figure 11B shows histograms after merging data from the three GPCR and four ion channel gene categories and offers a convenient and rather striking “pocket” summary: modal CA1 neurons co-express 19 distinct neuromodulatory GPCR genes and 65 distinct ion channel genes.

## Discussion

Several main findings emerge from the present analysis of single cell transcriptomes in area CA1 of mouse hippocampus. (1) A transcriptomic neurotaxonomy developed independently by genome-wide, function-agnostic clustering (Yao et al., 2021) captures highly diverse type-specific expression signatures of large numbers of genes encoding GPCRs, ion channel subunits and NPPs with remarkable precision. (2) Surprisingly large numbers (dozens) of different GPCR and ion channel genes are co-expressed at high levels in every CA1 neuron. (3) Abundant transcripts of one or more NPP genes are evident in nearly every CA1 neuron, suggesting that nearly every CA1 neuron is peptidergic as well as either GABAergic or glutamatergic. (4) For every one of the 18 NPP genes highly expressed in CA1, a cognate NP-GPCR is also highly expressed in the same area. Such pairing adds rodent hippocampus to the list of brain regions and species where RNA-Seq transcriptomics suggests the existence of densely multiplexed local peptidergic networks (Smith et al., 2019, 2020; Smith, 2021).

What might these findings have to say about how GPCR-mediated neuromodulation impacts CA1 network function? Most obviously, they suggest that the “hardware” is there to support highly multiplexed, highly vectorial modulatory signaling in CA1, with numerous specific modulators impacting numerous specific cells and cell types based on differential expression of genes encoding numerous receptors of highly differential ligand selectivity. Modulatory networks may thus embody highly intricate architectures shaped by diverse neuron-type-specific patterns of GPCR and NPP expression with diverse impacts upon membrane excitability and synaptic function governed by neuron-type-specific expression of ion channel genes. Some of these modulatory networks must involve ligands such as the monoamines and neuropeptides secreted by axons ramifying from distant brain regions, while others involve ligands such as peptides and endocannabinoids secreted by specific cell types nearby within CA1.

The large numbers of modulatory GPCRs expressed by every CA1 neuron (∼20) suggest that individual neurons must be parts of many overlapping but molecularly and architecturally distinct modulatory networks. These numbers alone indicate that a staggering combinatorial convergence of modulatory information must be accessible to each individual neuron (Smith et al., 2019; Taylor et al., 2021). To fully appreciate this potential information “bandwidth”, one must consider not only the number of GPCR genes in play, but also that modulatory responses are graded, or “analog,” with each GPCR’s agonist concentration, that subcellular GPCR localization surely matters, and that additional signaling diversity can be generated through physical and/or functional interactions when distinct GPCR protomers are co-expressed (Ferre et al., 2014; Kenakin, 2019).

Of course, all CA1 neurons are also parts of synaptic networks. Thus, CA1, must be viewed as a superimposition of synaptic and modulatory networks of comparable intricacy and neuron-type-specificity. Synaptic and modulatory connectivity vectors nonetheless surely differ in their origins, with synaptic connectivity governed solely by axonal and dendritic “wiring,” while modulatory connectivity, perhaps no less specifically, is governed as well by local ligand perfusion architectures, diffusion metrics and highly neuron-type-specific patterning of ligand and receptor gene expression. The fact that similar conclusion have been drawn based on various kinds of evidence from many other brain regions and species (Marder, 2012; Gjorgjieva et al., 2016) suggests that superimposition of synaptic and modulatory networks may be an absolute necessity for the adaptive function of CA1 hippocampus and perhaps all animal nervous systems. For instance, there have been suggestions that juxtaposition of recursively interacting synaptic and modulatory networks may be essential to nervous system capabilities as fundamental to animal survival as task learning and memory formation (Dayan, 2012; Gerstner et al., 2018; Moro et al., 2020; Liu et al., 2021).

Single-cell transcriptomes offer very useful hints as to what proteins may or may not be found in a given cell or cell type, but no simple proportionality between transcript and protein abundance can be assumed. Transcript abundance probably makes a loose prediction of a corresponding protein’s synthesis rate, but the actual abundance of that protein will still depend heavily upon the protein’s lifetime, which is known to vary quite widely amongst different proteins and cellular contexts, and on possible modulations of translation rate (Liu et al., 2016; Buccitelli and Selbach, 2020). Cell-level transcriptomes moreover offer no guidance at all as to subcellular protein localization. Subcellular localization of each must be critical to signaling from GPCRs to ion channels and therefore to neuromodulation. A substantial literature speaks to the likelihood that most or all GPCR and ion channel proteins are in fact localized to very specific subcellular regions (Lohse and Hofmann, 2015; Trimmer, 2015; Mykytyn and Askwith, 2017; Weinberg et al., 2019; Jullie et al., 2021), but particulars are lacking for most of these membrane proteins on most neurons. It is to be hoped that single-cell transcriptomes will help guide future investigations of both abundance and localization of neuromodulatory proteins.

Clearly scRNA-seq data in themselves provide no direct information about the morphology, electrophysiology or synaptic connectivity of a cell profiled, though such factors are obviously critical to understanding neuronal network structure and function. Molecular classifications nonetheless have a long history of neurobiological usefulness (Petilla Interneuron Nomenclature Group et al., 2008; DeFelipe et al., 2013; Fishell and Heintz, 2013; Kepecs and Fishell, 2014; Tremblay et al., 2016; Zeng and Sanes, 2017; Ibrahim et al., 2020). Transcriptomic neurotaxonomy currently offers the most promising “Rosetta Stone” to unite nominally disjunct information modalities and neuronal characteristics and many efforts to do so are well under way. Spatial transcriptomics are poised to soon provide accurate information about cell-type abundance (Zhuang, 2021). Type-specific transgenic animals and patch-seq experiments are already beginning to enable alignment of morphologies and electrophysiology with transcriptomic types (Gouwens et al., 2019, 2020; Lipovsek et al., 2021). Meanwhile, rabies tracing (Wall et al., 2016) and ambitious large-scale microscopy methods (Kleinfeld et al., 2011) promise to soon begin the integration of synaptic connectivity and cell type data. Synthesis of results from these disparate sources will no doubt be promoted strongly by emerging computational embedding methods (Gala et al., 2021).

Though very high-dimensional data such as those considered here often resist full interpretation from simple 2D visualizations like the present figures, there are indications that deeper exploration might reveal further interesting regularities. For example, we have already noted that some single-cell CPM distribution show outliers as marked in Figure 10B by arrows at low-CPM and by stars at high-CPM limits. Such outliers are most evident for genes encoding cell-cell signaling molecules and, interestingly, the arrows and stars coincide in most cases to mark the same histograms. Perhaps expression of these cell-cell signaling genes is driven by variable factors that do not impact expression of ion channel or G protein genes. Alternatively, still finer distinctions among stable cell types may be necessary to systematize such variations. Here, we’ll simply suggest the possibility that further analysis based on sophisticated dimensionality reduction methods may eventually prove rewarding.

Any ideas about nervous system function emerging from transcriptomic data can be taken only as hypothetical until subject to physiological test. Fortunately, the last decade has seen the growth of a truly remarkable new toolkit–heavy with fluorescence sensors of modulatory signaling and light-activated effectors applicable to live cells and behaving animals–that are rapidly transforming our capacities to test and refine hypotheses about cellular and networks impacts of neuromodulatory signaling. This new toolkit comprises sensors selective for modulatory ligands, reporters of GPCR and G protein activation, abilities to eavesdrop on numerous second-messenger systems and measure ion channel function. Here we can cite just a small sampling of an impressive decade’s landmarks and reviews (Banghart and Sabatini, 2012; Irannejad et al., 2013, 2014; Tsvetanova and von Zastrow, 2014; Spangler and Bruchas, 2017; Banghart et al., 2018; Patriarchi et al., 2019; Zeng et al., 2019; Ravotto et al., 2020; Sabatini and Tian, 2020; Smith et al., 2020; Stoeber et al., 2020; Unger et al., 2020; Jullie et al., 2021; Labouesse and Patriarchi, 2021; Redolfi et al., 2021; Tjahjono et al., 2021), while offering also a small sampling of progress emerging just at the time of this writing (Condon et al., 2021; Copits et al., 2021; Melzer et al., 2021; Wan et al., 2021; Duffet et al., 2022; Qian et al., 2022; Wu et al., 2022).

Many of the particulars of neuromodulatory transcriptomes we have outlined here for mouse hippocampus will certainly not apply directly to every other brain region in mouse and probably not in any exact way to hippocampus across other mammalian species. Even so, there are already indications of that many of the broad strokes we have painted here will generalize to other brain regions (Smith et al., 2019; Smith, 2021), other mammals including humans and even to non-mammalian tetrapods (Hodge et al., 2019; Bakken et al., 2021; Smith, 2021). We suggest here that the very high cell type specificity, multiplicity and diversity of GPCR, ion channel and NPP gene expression and co-expression we have just described will continue to surface as transcriptomes from more brain regions and species are similarly and even more deeply explored.

Finally, we’ll note that many of the neuromodulators we have focused upon here have drawn major drug development efforts. Several are targets of neuropsychiatric pharmaceuticals already in wide use and abuse (Wootten et al., 2013) and are often used in combination. It therefore seems reasonable to imagine that the coming of new information on the cell-type-specificity, multiplicity and diversity of modulator gene expression in human, like that we have portrayed for mouse, may contribute to meeting the clear and urgent need to define new molecular targets and therapeutic strategies (Hyman, 2012).

## Data Availability Statement

The original contributions presented in the study are included in the article/Supplementary Material, further inquiries can be directed to the corresponding author/s.

## Author Contributions

Both authors collaborated on conception, data analysis, and writing, contributed to the article, and approved the submitted version.

## Conflict of Interest

The authors declare that the research was conducted in the absence of any commercial or financial relationships that could be construed as a potential conflict of interest.

## Publisher’s Note

All claims expressed in this article are solely those of the authors and do not necessarily represent those of their affiliated organizations, or those of the publisher, the editors and the reviewers. Any product that may be evaluated in this article, or claim that may be made by its manufacturer, is not guaranteed or endorsed by the publisher.
