# Supplementary material for: A Molecular Landscape of Mouse Hippocampal Neuromodulation
Source: Front Neural Circuits. 2022 May 6;16:836930. doi: 10.3389/fncir.2022.836930 (PMC9120848; doi:10.3389/fncir.2022.836930)
Supplement: Supplementary file 1 [file Data_Sheet_1.ZIP › Supp_mats/Supplemental Figure 1.docx]

| 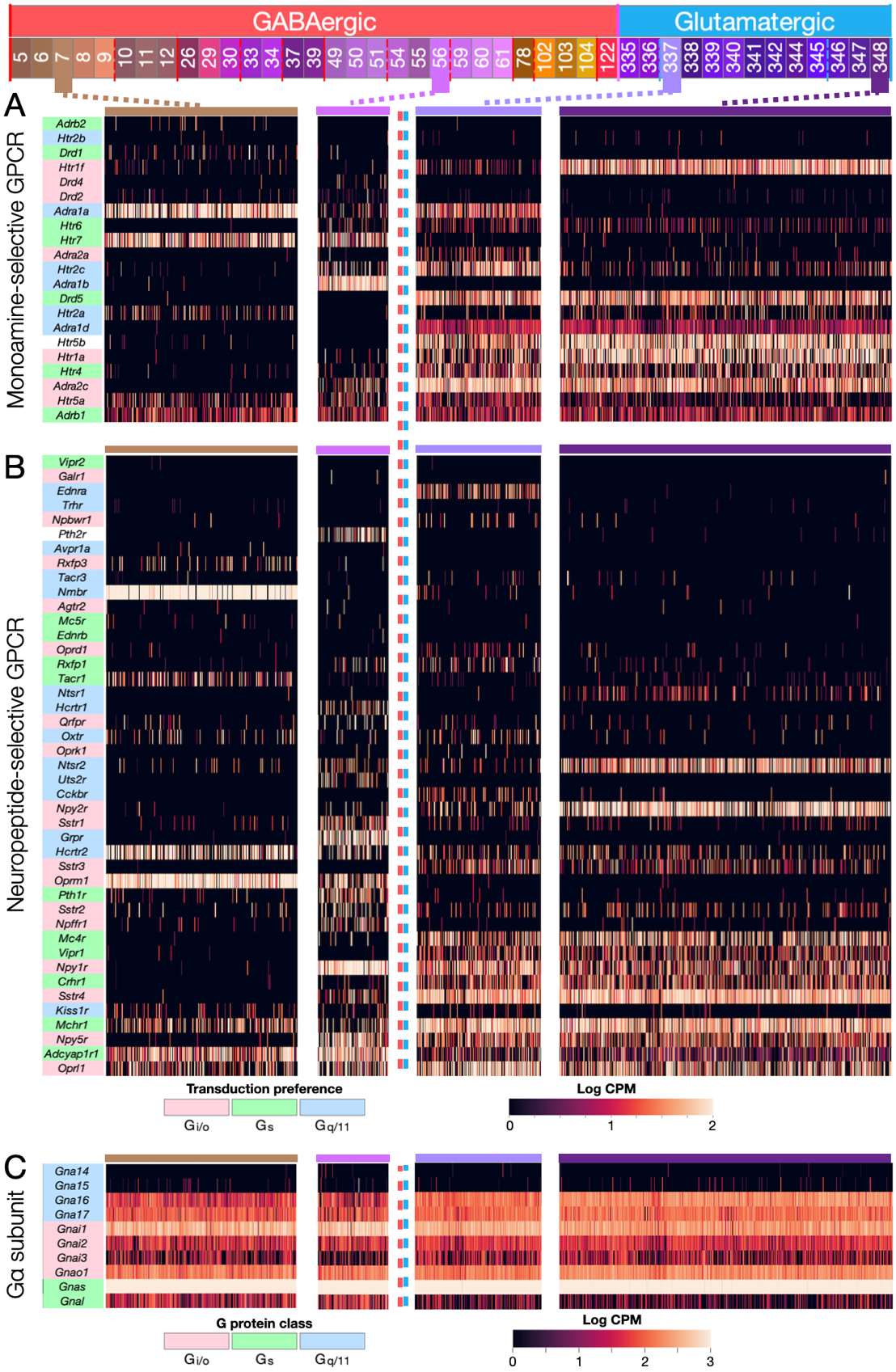 |
| --- |
| **Supplemental Figure 1.** *Single-cell expression signatures of 64 selected GPCR and 10 G protein genes in four CA1 cell types.* (A) Logarithmic heatmap representation of MA-GPCR transcript abundance across and within individual neurons of four selected CA1 cell types, as indicated in the text and defined according to the hierarchical neurotaxonomy summarized in Table 1. (B) A similar representation for NP-GPCRs. (C) A similar representation for G protein alpha subunits between and within the indicated CA1 cell types but using an extended intensity scale due to a generally higher level of G protein transcript expression relative to MA-GPCR and NP-GPCRs. |

Intrigued by hints that expression of some GPCR genes may juxtapose high type-specificity with comparatively high within-type variability, we have examined expression of 64 GPCR genes and 10 G protein α-subunit genes at the single-cell level more closely. Supplemental Figure 1 represents such expression for four representative, well-populated CA1 cell types – two GABAergic and two glutamatergic. The abundance of both particular MA-GPCR (A) and particular NP-GPCR (B) transcripts evident from the single cells is entirely consistent with the type-specificity evident from means in Figs. 3 and 4. These single-cell views nonetheless shed new light on the underlying within-type transcript-number distributions, suggesting that differential GPCR type-means may reflect different fractions of cells expressing GPCR genes, just as much as different amplitudes of expression by cells of a given type. Even for GPCR genes exhibiting high mean expression within a given type, many cells show very low expression. Conversely, some GPCR genes that show low mean expression for a given type show high expression in small fractions of cells.

Supplemental Figure 1C shows an interesting counterpoint: 10 Gα-subunit genes expressed at mean levels in the same range as the 64 GPCR genes expose very different single-cell expression profiles. In keeping with type-mean expression profiles as displayed in Fig. 6, expression of each of these 10 Gα-subunit genes shows very low differential between type clusters. Comparison of (A) and (B) with (C) also shows, however, much less variation within each of the four neuron types shown. These vignettes suggest that the expression of canonically neuromodulatory GPCR-encoding genes may be governed by stochastic factors or regulatory process not shared by all signaling genes.
