# Supplementary material for: A Molecular Landscape of Mouse Hippocampal Neuromodulation
Source: Front Neural Circuits. 2022 May 6;16:836930. doi: 10.3389/fncir.2022.836930 (PMC9120848; doi:10.3389/fncir.2022.836930)
Supplement: Supplementary file 1 [file Data_Sheet_1.ZIP › Supp_mats/Readme.docx]

Guide to Smith and von Zastrow 2022 Supplementary Materials

Materials in this directory permit generation of every data display item in the parent publication from previously published single-cell RNA-Seq datasets (Yao, et al. 2021 *Cell* 184, 1–20: "Yao21" here). Besides this pdf, the unzipped directory structure should include two Excel spreadsheets, eight Jupyter notebook python scripts, one folder "Keys/" containing two small CSV files and two other initially empty folders, "Resources/" and "Products/". Table 1 and all data figures (Figs. 1-11) are composed from screen shots off the spreadsheet "Data_display.xlsx" (which also includes copious annotation). This spreadsheet offers access to all parent publication data display items in numerical as well as graphical form and extends both treatments from our small "vignettes" to all 1,749 of the candidate CA1 neuromodulation genes studied. The macro-enabled spreadsheet "Mapper.xlsm", the python scripts and key files listed below provide for the reconstruction of all displayed data from three primary data resource files.

The Yao21 resource files tabulated below are available for download from an Allen Institute web page: (<https://portal.brain-map.org/atlases-and-data/rnaseq/mouse-whole-cortex-and-hippocampus-smart-seq>).

Primary Resource Files:

| "Exons only" (gene X cell matrices) | expression_matrix_exons.csv | 7,363,207,437 bytes |
| --- | --- | --- |
| "Introns only" (gene X cell matrices) | expression_matrix_introns.csv | 6,905,937,488 bytes |
| "Table of cell metadata" (metadata for each cell) | metadata.csv | 29,481,556 bytes |

Download these three resource files and move them into the "Resources/" folder. Scripts have been tested using the Anaconda MacOS distribution of Jupyter Notebook, versions 6.3.0 and later. 8 GB of memory should be more than adequate to execute these scripts: the working directory will grow to ~18 GB of storage as the Python scripts populate the "Products" folder with a cascade of eleven CSV sequentially dependent files. The numbered Python scripts must be executed initially in the order indicated by their file names and the table below and it is critical to allow each to finish before launching the next. The present files expose only a small subset of the mouse brain regions, cell types and genes in the Yao21 SMART-Seq isocortex/hippocampal dataset. With some relatively minor editing, these scripts should be useful for other deeper and/or broader explorations of the voluminous Yao21 dataset. One additional Python script, "Downsampler", is provided to allow exploration of mean signature resistance to random downsampling.

Jupyter Notebook Python Scripts:

|  | Script | Input Files | Product Files |
| --- | --- | --- | --- |
| 1 | Tally_sample_reads | expression_matrix_exons.csv,  expression_matrix_introns.csv | Mapped_exon_reads.csv, Mapped_intron_reads.csv,  Mapped_reads.csv |
| 2 | Sift_comm_genes | expression_matrix_exons.csv,  expression_matrix_introns.csv, Mouse_Comm_sifter.csv | Comm_exon_counts.csv, Comm_intron_counts.csv |
| 3 | Sum_exons_introns | Comm_exon_counts.csv, Comm_intron_counts.csv | Comm_summed_counts.csv |
| 4 | Counts_to cpms | Comm_summed_counts.csv, Mapped_reads.csv | Comm_cpms.csv |
| 5 | Merge_annos_cpms | metadata.csv, Comm_cpms.csv | Comm_annos_cpms.csv |
| 6 | Select_regions | Comm_annos_cpms.csv | Comm_HIP_annos_cpms.csv |
| 7 | Select_clusters | Comm_HIP_annos_cpms.csv, CA1_clusters.csv |  |
| 8 | Cluster_means | Comm_CA1_comms.csv | Comm_CA1_comm_means.csv |

Key Files:

| Mouse_comm_genes.csv | List of 1,749 selected communication genes |
| --- | --- |
| CA1_Clusters.csv | List of all 42 HIP-CA1 Yao21 clusters with >16 samples |
